# Catenin Alpha-2 Mutation Changes the Immune Microenvironment in Lung Adenocarcinoma Patients Receiving Immune Checkpoint Inhibitors

**DOI:** 10.3389/fphar.2021.645862

**Published:** 2021-06-07

**Authors:** Yang Wen, Anqi Lin, Weiliang Zhu, Ting Wei, Peng Luo, Linlang Guo, Jian Zhang

**Affiliations:** ^1^ Department of Oncology, Zhujiang Hospital, Southern Medical University, Guangzhou, China; ^2^ Department of Pathology, Zhujiang Hospital, Southern Medical University, Guangzhou, China

**Keywords:** CTNNA2, lung adenocarcinoma, immune checkpoint inhibitors, immune microenvironment, prognosis

## Abstract

**Background:** Lung cancer has always been the most prevalent cancer. Lung adenocarcinoma (LUAD) is the most common lung cancer subtype and has a high tumor mutation rate. In addition to KRAS, EGFR, ALK, HER2, ROS1, and BRAF, which are known to have high mutation rates, we discovered some new mutated genes, such as catenin alpha-2 (CTNNA2), in LUAD patients treated with immune checkpoint inhibitors (ICIs). These mutant genes are potential therapeutic targets for LUAD.

**Methods:** We analyzed a cohort of LUAD patients with somatic mutation and survival data in the Cancer Genome Atlas (TCGA) database and a cohort of LUAD patients receiving immune checkpoint inhibitors with clinical data and whole-exome sequencing (WES) mutation data to evaluate the role of CTNNA2 gene mutation in LUAD. In addition, CIBERSORT was used to analyze the immune characteristics of CTNNA2 wild-type patients and CTNNA2 mutant-type patients, and gene set enrichment analysis (GSEA) was employed for pathway enrichment analysis. The results were verified by downloading data regarding the drug sensitivity of LUAD cell lines from the Genomics of Drug Sensitivity in Cancer (GDSC) database.

**Results:** We found that CTNNA2 mutation was associated with longer overall survival (OS) in LUAD patients. Analysis of the cohort from the Cancer Genome Atlas showed that patients with CTNNA2 mutation had more tumor neoantigens and a greater tumor mutation burden (TMB). Through further analysis of the tumor immune microenvironment, we found that in LUAD patients with CTNNA2 mutations, the gene expression levels of chemokine C-X-C motif chemokine 9 (CXCL9) and granzyme B (GZMB) were elevated, and the gene expression level of inhibitory receptor killer cell immunoglobulin-like receptor 2DL1 (KIR2DL1) was significantly reduced. These alterations might affect gene expression in macrophages, NK cells, and mast cell markers. In addition, LUAD patients with CTNNA2 mutation had a significantly increased number of mutations in DNA damage response (DDR) genes. The drug susceptibility results and gene set enrichment analysis showed that after CTNNA2 mutation occurred, changes were found in the DNA damage response pathway, the phosphoinositide 3-kinase (PI3K) pathway and others, indicating that CTNNA2 mutation can regulate the activation of PI3K and DDR pathways.

**Conclusion:** Our findings provide novel insights into the underlying pathogenesis of LUAD. CTNNA2 mutation can change the immune microenvironment, thereby improving patient prognosis. The results also suggest that CTNNA2 may become a new biomarker and therapeutic target for LUAD in the future.

## Introduction

Globally, lung cancer is the leading cause of cancer-related death (Nasim et al., 2019). Lung cancer includes small cell lung cancer and non-small cell lung cancer (NSCLC), and NSCLC accounts for 85% of all lung cancers. NSCLC is divided into different histological types, including adenocarcinoma, squamous cell carcinoma, large cell carcinoma, adenosquamous carcinoma, sarcomatoid carcinoma, etc. Among them, adenocarcinoma accounts for more than 50% of NSCLCs (Gridelli et al., 2015), and lung adenocarcinoma (LUAD) has become the most common type of lung cancer. Common mutation sites driving oncogenes in LUAD are KRAS (25%), EGFR (21%), ALK (7%), MET (3%), HER2 (2%), ROS1 (2%), BRAF (2%), RET(2%), NTRK1(1%), PIK3CA(1%), MEK1(1%), and other unknown oncogenic drivers (31%) (Hirsch et al., 2017; Inamura, 2018). Using drugs with more specific targets can improve the prognosis of patients, and immunotherapy has become one of the most promising treatment strategies (Gridelli et al., 2015; Hirsch et al., 2017).

Currently, the most studied immune checkpoints in lung cancer mainly include cytotoxic T lymphocyte-associated antigen 4 (CTLA-4), programmed cell death receptor 1 (PD-1), and programmed cell death receptor ligand 1 (PD-L1), etc (Gubin et al., 2014; Steven et al., 2016). Immune checkpoint inhibitors mainly activate T cells by blocking these molecules and exert antitumor effects. Monoclonal antibody-based therapies targeting CTLA-4 and/or PD-1 (checkpoint block) have produced significant clinical benefits for patients with different malignancies (Hodi et al., 2010; Wolchok et al., 2013). The immune microenvironment plays an important role in the regulation of immunotherapy response (Menzel and Black, 2020). Tumors are complex tissues composed of not only tumor cells but also stromal cells, inflammatory cells, vasculature and extracellular matrix (ECM). The sum of all of these factors is defined as the tumor microenvironment (Quail and Joyce, 2013). Although the success of immunotherapy is exciting and countless patients have achieved remarkable results with immunotherapy treatment, there are still some patients who do not respond to immunotherapy (Suresh et al., 2018). As technology has advanced, the complexity and diversity of the tumor microenvironment and its important impact on immunotherapy have been revealed. Further analysis and understanding of the tumor immune microenvironment will help improve the response to immunotherapy.

Alpha-catenin is a mechanosensing protein that undergoes conformational changes under the action of cytoskeletal tension, thereby changing the connection between cadherin and the actin backbone (Vite et al., 2015). There are three α-catenin subtypes in mice and humans: CTNNA1 (αE-catenin, epithelial), CTNNA2 (αN-catenin, nerve) and CTNNA3 (αT-catenin, testis) (Vite et al., 2015). CTNNA2 is related to the development of the nervous system and many neurological diseases (Takeichi and Abe, 2005). Recent articles have shown that CTNNA2 is also involved in the occurrence and development of some tumors. According to reports, CTNNA2 mutation is involved in the adhesion junction pathway, which is one of the most disturbed pathways in gastric cancer (Wang et al., 2014). CTNNA2 and CTNNA3 are also frequently mutated tumor suppressor genes in laryngeal cancer (Fanjul-Fernandez et al., 2013). In addition, CTNNA2 mutation is associated with the prognosis of patients with pancreatic ductal adenocarcinoma (PDAC) (Rizzato et al., 2016). However, the role of CTNNA2 mutation in lung cancer has not been studied, and the relationship between the tumor microenvironment and CTNNA2 mutation remains unknown.

In this article, we analyzed data from The Cancer Genome Atlas LUAD cohort, an ICI-treated cohort, and LUAD cell line drug sensitivity data from the Genomics of Drug Sensitivity in Cancer (GDSC) database to elucidate the clinical and immune characteristics of LUAD patients with CTNNA2 mutation. The results suggest that CTNNA2 can be used as a prognostic target for LUAD patients.

## Materials and Methods

### Catenin Alpha-2 Gene Mutation and the Prognosis of Lung Adenocarcinoma Treated with Immunotherapy

Clinical data and WES mutation data on the immunotherapy cohort (Miao et al., 2018) were collected to evaluate the relationship between CTNNA2 mutation and the prognosis of LUAD patients receiving immune checkpoint inhibitors (ICIs). According to CTNNA2 mutation status, we grouped LUAD patients who received ICI treatment (anti-PD-1/PD-L1/anti-CTLA-4 therapies) and had mutation data (*n* = 47) into CTNNA2 mutant-type (MT) and CTNNA2 wild-type (WT) groups and then performed Kaplan-Meier (KM) analysis. In addition, we used the R package TCGAbiolinks (Colaprico et al., 2016) to download the somatic mutation and survival data (overall survival) of the LUAD cohort from TCGA in Genomic Data Commons (https://portal.gdc.cancer.gov/). cBioportal (Cerami et al., 2012) was used to download TCGA-LUAD survival data (disease-free survival), and KM analysis of TCGA-LUAD patient survival was performed according to the mutation status of the CTNNA2 gene.

### Gene Mutation Characteristics

Targeted next-generation sequencing (NGS; MSK-IMPACT) was used by Miao et al. to report the somatic mutation data in 47 LUAD samples. In addition, many papers in the LUAD cohort of TCGA have analyzed neoantigen loading (NAL) data (Thorsson et al., 2019).

### Immune Characteristics and Tumor Immunogenicity

We downloaded the gene expression data (Illumina HiSeq, RNASeq) of the LUAD cohort of TCGA from TCGAbiolinks, and used CIBERSORT (Newman et al., 2015) for the analysis. We mainly assessed the infiltration of 22 immune cell types in the CTNNA2-WT group and CTNNA2-MT group in the LUAD cohort of TCGA (|logFC| > 1, *p* < 0.05). In addition, the mRNA expression of immune-related genes in the CTNNA2-WT group and CTNNA2-MT group in the LUAD cohort of TCGA (|logFC| > 1, *p* < 0.05) was also analyzed. We obtained immune cell-related genes from the existing literature (Hao et al., 2018), and quantified the expression level of these genes as log2 (FPKM + 1). Among them, immune-related genes and their functional classification and immune-related scores have been reported in the literature (Thorsson et al., 2019). To better illustrate the gene mutation characteristics, the R package ComplexHeatmap (Gu et al., 2016) was used to visualize the top 20 gene mutations and clinical features of the ICI-treated cohort and the LUAD cohort of TCGA.

### Pathway Enrichment Analysis

TCGAbiolinks was used to download the gene expression data (raw count) of TCGA-LUAD, and difference analysis was performed using the R package edgeR (Robinson et al., 2010). The ClusterProfiler R package (Yu et al., 2012) was used for gene annotation enrichment analysis. In addition, this article uses the GSEA gene set in the MSigDB database of the Broad Institute (Subramanian et al., 2005). In terms of statistics, *p* < 0.05 was considered a significant difference for Gene Ontology (GO) terms and Kyoto Encyclopedia of Genes and Genomes (KEGG) and Reactome pathway databases.

### Drug Sensitivity Analysis

This article uses the LUAD cell line with WES data for analysis. The specific information of the cell line was obtained from the GDSC database (Yang et al., 2013). In addition, the nonsynonymous mutations were taken as the raw mutation count and divided by 38 Mb to quantify the tumor mutation burden (TMB) in the ICI-treated cohort, the LUAD cohort of TCGA and the GDSC-LUAD cohort. This calculation method is consistent with that published in the literature (Chalmers et al., 2017). The information downloaded from the GDSC database included the susceptibility data of the LUAD cell line for comparisons of the sensitivity of CTNNA2-WT and CTNNA2-MT cell lines to different chemotherapeutic drugs and molecular targeted drugs.

### DNA Damage Response and Repair Pathway Mutation Number Analysis

We evaluated the number of nonsynonymous mutations in the DDR pathway in the ICI-treated cohort, TCGA-LUAD cohort and GDSC-LUAD cohort and compared the number of nonsynonymous mutations in the DDR pathway between the CTNNA2-WT group and the CTNNA2-MT group. For these analyses, we used the DDR pathway gene set of the Molecular Signatures Database (MSigDB) of the Broad Institute (Subramanian et al., 2005), see Additional File: Supplementary Table S1 for details.

### Copy Number Variation Analysis

The data come from the Affymetrix SNP 6.0 microarray (hg19; germline/potential false-positive calls were removed) of the LUAD cohort of TCGA, downloaded through the Broad GDAC Firehose tool (http://gdac.broadinstitute.org/), and the data are saved in CNV segment format. The downloaded data were analyzed by GISTIC2.0, and the analysis platform was GenePattern (Reich et al., 2006) (https://cloud.genepattern.org/gp/pages/index.jsf). In terms of GISTIC2.0 analysis parameter settings, the confidence level was 0.99; the X-chromosome was not excluded before analysis, and the rest of the analysis uses default settings. GISTIC2.0 uses R package Maftools (Mayakonda et al., 2018) to visualize CNVs.

### Statistical Analysis

Progression-free survival (PFS) was defined as the time from the introduction of ICIs to progression on ICIs. Overall survival (OS) was defined as the time from the introduction of ICI to death. The Mann–Whitney U test was used to compare the abundance of immune cells, the expression of immune-related genes, the number of gene mutations in the DDR pathway, and the drug sensitivity in the GDSC database between the CTNNA2-WT group and the CTNNA2-MT group. Fisher’s exact test was used to compare sex, age, OS, PFS, TMB, smoking history, treatment response and the mutation status of the top 20 genes between the CTNNA2-WT group and the CTNNA2-MT group in the immunotherapy cohort. The chi-square test was used to compare the difference in tobacco smoking history in the LUAD cohort of TCGA between the CTNNA2-WT group and the CTNNA2-MT group. Fisher’s exact test was used to compare the mutation status of the top 20 genes in the LUAD cohort of TCGA between the CTNNA2-WT group and the CTNNA2-MT group and to compare sex, race, ethnicity, Neoantigens, OS, TMB, pack-years and clinical stage in the LUAD cohort of TCGA between the CTNNA2-WT group and the CTNNA2-MT group. The Kaplan-Meier method was used to estimate the PFS and OS of the entire cohort, and the log-rank test was used for survival analysis. Differences with *p* < 0.05 were considered statistically significant, and all statistical tests were two-sided tests. All statistical tests and visual analysis were performed in R software (version 3.6.1). In addition, the R package ggpurb is used to visualize box plots. Differential analyses were visualized via the “ggpubr” package (https://CRAN.R-project.org/package=ggpubr). The false discovery rate (FDR) cutoff for CNV visualization was 0.05.

## Results

### Catenin Alpha-2 Gene Mutation was Associated with Clinical Features of Patients with Lung Adenocarcinoma

**FIGURE 1:**
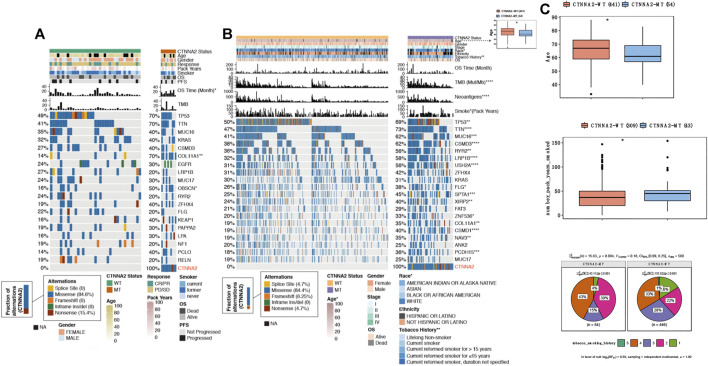
The clinical features of patients with CTNNA2-WT LUAD and CTNNA2-MT LUAD. **(A)** The heat map shows the differences in the mutation status of the top 20 genes and in sex, age, OS, PFS, TMB, smoking history and treatment response between the CTNNA2-WT (*n* = 37) and CTNNA2-MT (*n* = 10) LUAD patients in the immunotherapy cohort (ICI-treated). The data came from the Memorial Sloan-Kettering Cancer Center (MSKCC). **(B)** The heat map shows the differences in the mutation status of the top 20 genes and in sex, race, tumor neoantigens, OS, TMB, pack-years and clinical stage between the CTNNA2-WT (*n* = 459) and CTNNA2-MT (*n* = 55) LUAD patients in the LUAD cohort of TCGA. **(C)** Box plot of the age of CTNNA2-WT (*n* = 441) and CTNNA2-MT (*n* = 54) LUAD patients in the LUAD cohort of TCGA. Fisher’s exact test, *p* < 0.05. **(D)** Box plot of the age of CTNNA2-WT (*n* = 441) and CTNNA2-MT (*n* = 54) LUAD patients in the LUAD cohort of TCGA. Fisher’s exact test, *p* < 0.05. **(E)** The pie chart comparing the tobacco smoking history of CTNNA2-WT (*n* = 446) and CTNNA2-MT (*n* = 54) LUAD patients in the LUAD cohort of TCGA. Chi-square test, Pearson χ2 (4) = 15.63, *p* = 0.004, V Cramer = 0.18, 95% CI [0.09, 0.25], nobs = 500.

We used an immunotherapy cohort with clinical data and WES mutation data to evaluate the relationship between CTNNA2 mutation and the prognosis of LUAD patients receiving ICIs (see *Materials and Methods*) (Figure 1A). This cohort included 10 CTNNA2-MT patients and 37 CTNNA2-WT patients. We found that compared with CTNNA2-WT patients, CTNNA2-MT patients had a longer OS time (log-rank test, *p* < 0.05). In addition, missense mutation was the most common mutation mode in CTNNA2-MT patients, which accounted for 84.6%, followed by nonsense mutation, which accounted for 15.4%. CTNNA2-MT patients often had other gene mutations, the most common of which were COL11A1 and OBSCN mutations. The mutation frequency increased for some other genes as well, such as TP53, TTN, MUC16, and KRAS. In addition, we analyzed factors such as sex, age, pack years, response, smoking, PFS, TMB, etc., but no difference was found between the two groups. The top 30 mutant genes of ICI-treated cohort are shown in Supplementary Figure S1.

We also downloaded the LUAD cohort of TCGA survival data (see *Materials and Methods*, Figure 1B). A total of 514 LUAD patients were in this cohort, including 55 CTNNA2-MT patients and 459 CTNNA2-WT patients. We found that compared with CTNNA2-WT patients, CTNNA2-MT patients had a significantly higher TMB and more tumor neoantigens (Mann-Whitney U test, *p* < 0.05, Figure 1B). Missense mutation was the most common mutation mode of CTNNA2-MT patients, which accounted for 84.4%. Other mutation modes included splice site, frame shift, and nonsense mutations. The proportion of CTNNA2-MT patients with other gene mutations, such as TP53, TTN, MUC16, and CSMD3, was higher than that of CTNNA2-WT patients (Fisher test, *p* < 0.05, Figure 1B). The average age of CTNNA2-MT patients was lower than that of CTNNA2-WT patients (Fisher test, *p* < 0.05, Figure 1C), and differences in ethnic distribution were found (Fisher test, *p* = 0.029). CTNNA2 mutation was more common in African Americans than Caucasians. Patients with CTNNA2 mutation generally smoked longer (Fisher’s exact test, *p* < 0.05, Figure 1D), and CTNNA2-MT patients also had more pack-years than CTNNA2-WT patients (Pearson χ2 4) = 15.63, *p* = 0.004, Figure 1E). In addition, we analyzed clinical stage and OS, but no difference was observed between the two groups. Based on the above two cohort studies, we found that the clinical differences between the two groups were obvious and that CTNNA2-MT patients had a longer overall survival than CTNNA2-WT patients.

### Immune Microenvironment Characteristics of Catenin Alpha-2 Mutant-Type Patients in the Checkpoint Inhibitor-Treated Cohort and Cancer Genome Atlas-Lung Adenocarcinoma Cohort

**FIGURE 2:**
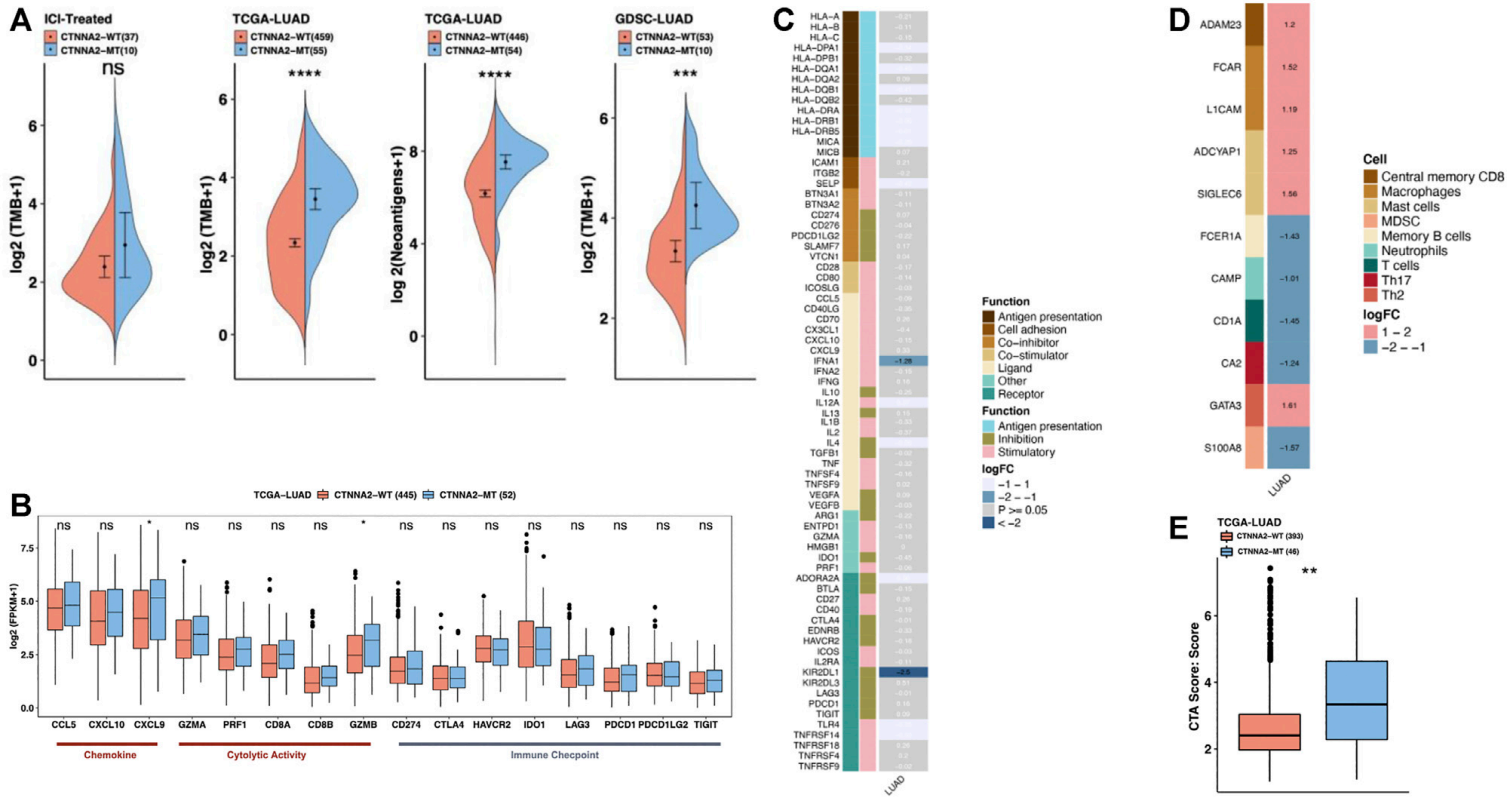
The immune microenvironment characteristics of CTNNA2-MT patients. **(A)** The first chart shows the relative expression of TMB in CTNNA2-WT (*n* = 37) and CTNNA2-MT (*n* = 10) LUAD patients in the ICI-treated cohort. The second chart shows the relative TMB expression of CTNNA2-WT (*n* = 459) and CTNNA2-MT (*n* = 55) LUAD patients in the LUAD cohort of TCGA. The third chart shows the relative expression of neoantigens of CTNNA2-WT (*n* = 446) and CTNNA2-MT (*n* = 54) patients in the LUAD cohort of TCGA. The fourth chart shows the relative expression of TMB in the CTNNA2-WT (*n* = 53) and CTNNA2-MT (*n* = 10) LUAD cell lines in the GDSC database. Mann-Whitney U test, ns means no statistical significance, *means *p* < 0.05; **means *p* < 0.01; ***means *p* < 0.005; and ****means *p* < 0.001. **(B)** The immune gene box diagram shows the relative expression levels of 16 immune genes (chemokines/cytolytic activity/immune checkpoints) in patients with LUAD of CTNNA2-WT (*n* = 445) and CTNNA2-MT (*n* = 52) in the LUAD cohort of TCGA. Mann–Whitney U test, ns means no statistical significance, *means *p* < 0.05. **(C)** The immune-related gene heat map shows the relative expression of 74 immune-related genes (antigen presentation/stimulatory/inhibitory) in patients with CTNNA2-WT and CTNNA2-MT LUAD in the LUAD cohort of TCGA. The white font represents the genes that were not differentially expressed, and the row annotation in the heat map represents the function of the gene; see Methods for details. **(D)** The heat map of immune cell-related genes shows the relative expression of 11 immune cell-related genes in patients with CTNNA2-WT and CTNNA2-MT LUAD in the LUAD cohort of TCGA. The row annotation in the heat map represents the cell type from which the gene originated. **(E)** The CTA immune score of CTNNA2-WT (*n* = 393) and CTNNA2-MT (*n* = 46) patients in the LUAD cohort of TCGA.

Clinical data initially showed that in LUAD, CTNNA2 mutation was associated with high TMB (Figure 1B). High TMB can help predict the efficacy of tumor immunotherapy in patients with lung cancer, bladder cancer, and melanoma, and TMB is an important and independent predictive biomarker. Analysis of the GDSC database showed that LUAD cells with CTNNA2 mutation had a higher TMB than CTNNA2-WT patients (Mann-Whitney U test, *p* < 0.05 Figure 2A), and analysis of the cohort of TCGA verified this result. The ICI-treated cohort also exhibited this trend. More importantly, the analysis of the cohort of TCGA showed that CTNNA2-MT patients had more tumor neoantigens than CTNNA2-WT patients (Mann-Whitney U test, *p* < 0.05 Figure 2A). Studies have shown that patients with advanced non-small-cell lung cancer who had more tumor antigens were more sensitive to immunotherapy (Rolfo et al., 2017), which suggests that LUAD patients with CTNNA2 mutation may be more likely to benefit from immunotherapy than those without this mutation.

Chemokines can induce chemotaxis and promote the differentiation of immune cells; thus, their anticancer effects are worthy of further study. We analyzed the relative expression of 16 immune genes (chemokines/cytolytic activity/immune checkpoints) in CTNNA2-WT and CTNNA2-MT patients in the LUAD cohort of TCGA. After CTNNA2 mutation, CXCL9 and GZMB gene expression levels were found to increase (Mann-Whitney U test, Figure 2B). Evidence has shown that the CXCL9 axis can not only activate the antitumor activity of immune cells (Tokunaga et al., 2018) but can also play an important role in the response to immune checkpoint inhibitors (Zhang et al., 2018). Moreover, CXCL9 can exert a tumor suppressive function in tumors (Bronger et al., 2016). This suggests that CXCL9 can exert antitumor effects by affecting other immune cells in LUAD. However, the role of GZMB in immunotherapy is unclear, and CTNNA2 mutation had no significant effect on the gene expression of immune checkpoints. CD274 and LAG3 exhibited an increasing trend, while IDO1 exhibited a decreasing trend.

We also analyzed the relative expression of 74 immune-related genes and 11 immune cell-related genes in patients with CTNNA2-WT and CTNNA2-MT in the LUAD cohort of TCGA (|logFC| > 1, *p* < 0.05), and the results showed that the gene expression level of inhibitory receptor KIR2DL1 decreased most significantly after CTNNA2 mutation occurred (Figure 2C). KIR2DL1 is the receptor for some HLA-C alleles on natural killer cells, which can inhibit the activity of NK cells, thereby preventing cell lysis (Hilton and Parham, 2017). In patients with CTNNA2 mutation, the expression level of KIR2DL1 was reduced, and the factors that prevent cell lysis were reduced, leading to cell death. CTNNA2 mutation has been shown to affect immune cells, with different immune cells exhibiting different genetic changes. After CTNNA2 mutation, the gene expression level of FCER1A in central memory B cells, CAMP in neutrophils, CD1A in T cells, CA2 in Th17 cells, and S100A8 in myeloid-derived suppressor cells (MDSCs) were found to decrease (Figure 2D). Furthermore, the gene levels of a disintegrin and metalloprotease 23 (ADAM23) in central memory B cells, Fc fragment of IgA receptor (FCAR) and L1 cell adhesion molecule (L1CAM) in macrophages, adenylate cyclase activating polypeptide 1 (ADCYAP1) and sialic acid binding Ig-like lectin 6 (SIGLEC6) in mast cells, and GATA binding protein 3 (GATA3) in Th2 cells were found to increase (Figure 2D). Among them, ADAM23 is considered to be a possible tumor suppressor gene and is often downregulated in various malignant tumors (Zmetakova et al., 2019). In addition, we used the cancer testis antigen (CTA) immune scoring system to score CTNNA2-WT and CTNNA2-MT LUAD patients in the LUAD cohort of TCGA (see *Materials and Methods*). The results confirmed that CTNNA2-MT LUAD patients had higher CTA scores (Figure 2E). These results indicate that CTNNA2 mutation may stimulate the increase in gene expression in a series of immune cells by increasing the expression of chemokine CXCL9, thereby enhancing the immune response of patients with LUAD.

### The Number of DNA Damage Response Pathway Mutations was Higher in Catenin Alpha-2 Mutant-Type Patients with Lung Adenocarcinoma

**FIGURE 3:**
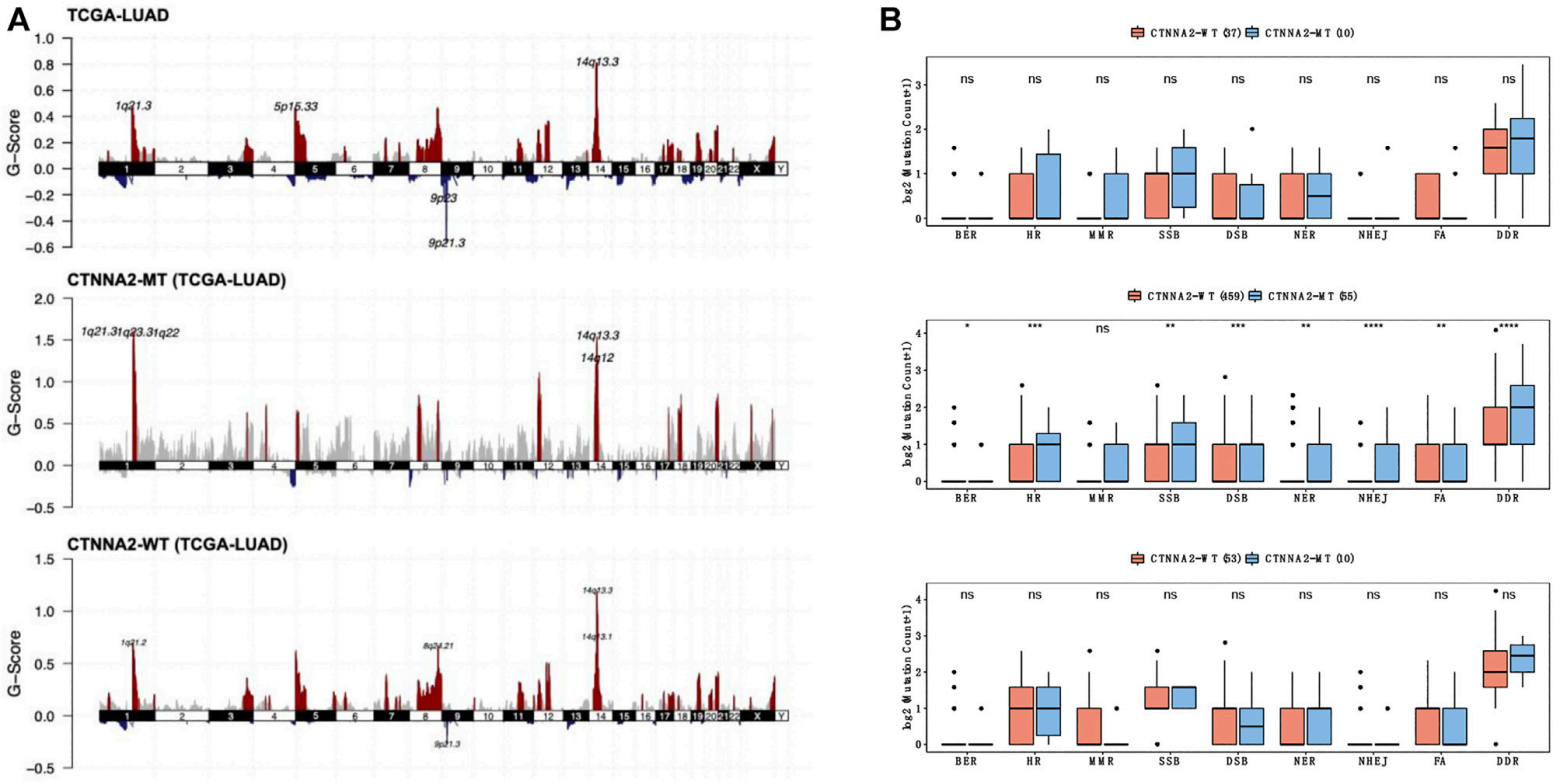
The differences in DDR pathway mutations between the CTNNA2-MT group and the CTNNA2-WT group. **(A)** The copy number variation in CTNNA2 in the LUAD cohort of TCGA; red represents an increase in copy number fragments, blue represents a loss of copy number fragments, and the mutation sites have been marked. **(B)** The picture above shows the number of nonsynonymous mutations in DDR-related pathways in CTNNA2-WT patients (*n* = 37) and CTNNA2-MT patients (*n* = 10) in the ICI-treated cohort. The middle picture shows the number of nonsynonymous mutations in DDR-related pathways in CTNNA2-WT patients (*n* = 459) and CTNNA2-MT patients (*n* = 55) in the LUAD cohort of TCGA. The picture below shows the number of nonsynonymous mutations in the DDR-related pathways of CTNNA2-WT (*n* = 53) and CTNNA2-MT (*n* = 10) LUAD cell lines in the GDSC database. Mann–Whitney U test, ns means no statistical significance; *means *p* < 0.05; **means *p* < 0.01; ***means *p* < 0.005; and ****means *p* < 0.001.

To describe the specific situation of CTNNA2 mutation, we detected the copy number variation of CTNNA2 gene mutation sites among LUAD patients in TCGA (Figure 3A), and we found that CTNNA2-WT patients had an increased copy number of 14q13.3, 14q13.1, 8q24.21, and 1q21.2, and a decreased copy number of 9p21.3. CTNNA2-MT patients had an increased copy number of 14q13.3, 14q12, 1q21.3, 1q23.3, and 1q22. The G-score of CTNNA2-MT patients at these common mutation sites was higher than that of CTNNA2-WT patients. In addition, we compared the number of nonsynonymous mutations in DDR-related pathways between CTNNA2-WT and CTNNA2-MT patients in the ICI-treated cohort and LUAD cohort of TCGA. The same analysis was performed on LUAD cell lines in the GDSC database. The list of genes included in the DDR genome used for comparative analysis is shown in Supplementary Table S1. The results showed that in the LUAD cohort of TCGA, there was a significant increase in DDR mutations among CTNNA2-MT patients (Mann-Whitney U test, *p* < 0.05, Figure 3B). DDR analysis of the other two databases showed the same trend. According to the analysis of the LUAD cohort of TCGA, the number of nonsynonymous mutations in CTNNA2-MT patients increased mainly in three pathways: homologous recombination (HR), double strand break (DSB), and nonhomologous end joining (NHEJ). These results suggest that CTNNA2-MT patients with LUAD have more mutations in the DDR pathway, and these mutation targets are not only targets for immunotherapy but also suggest a favorable prognosis (Pilié et al., 2019).

### Catenin Alpha-2 Mutation was Related to the Drug Sensitivity of Some Targeted Drugs

**FIGURE 4:**
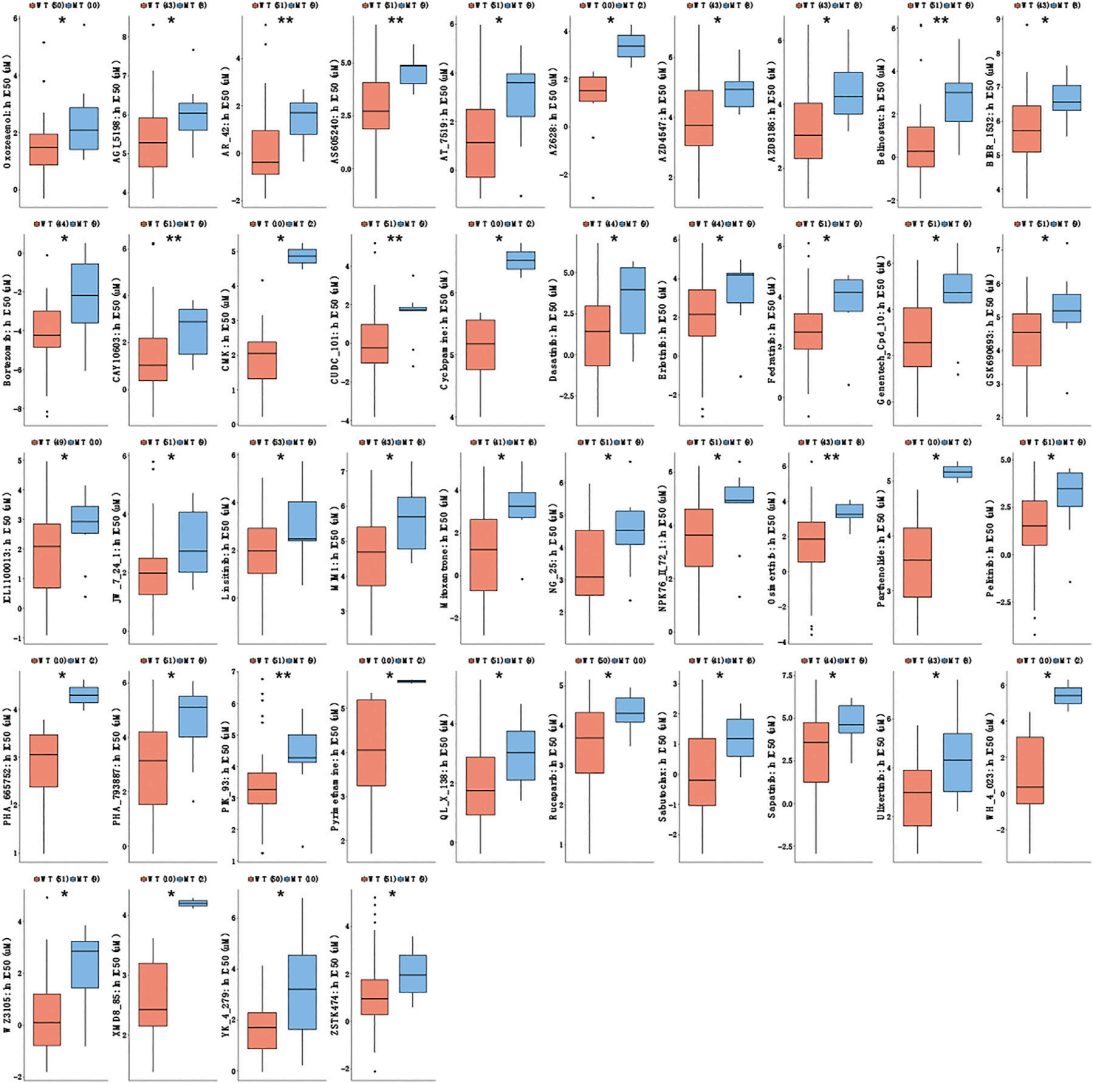
Drug sensitivity results of CTNNA2-MT LUAD cell lines. The box plot shows the IC50 of CTNNA2-WT and CTNNA2-MT LUAD cell lines for targeted drugs in the GDSC database; the unit of measurement is μM. Mann–Whitney U test, ns means no statistical significance; *means *p* < 0.05; **means *p* < 0.01.

We used the GDSC database to analyze the sensitivity of CTNNA2-WT and CTNNA2-MT LUAD cell lines to some drugs. The results confirmed that the half-maximal inhibitory concentration (IC50) of eight common chemotherapy drugs, namely, cisplatin, docetaxel, etoposide, gemcitabine, irinotecan, paclitaxel, pemetrexed and vinorelbine, tended to be higher in CTNNA2-MT LUAD cells than in WT cells (Supplementary Figure S2). We also used the GDSC database to analyze the sensitivity of CTNNA2-WT and CTNNA2-MT LUAD cell lines to EGFR-TKI and ALK inhibitors commonly used in clinical practice. The results confirmed that the half-maximal inhibitory concentration (IC50) of erlotinib was higher in CTNNA2-WT LUAD cell lines, with no difference between the two groups with regard to other inhibitors (Supplementary Figure S3). Interestingly, we further tested 351 drugs and found that in CTNNA2-MT LUAD cell lines, the IC50 of 44 drugs increased, indicating that the CTNNA2-MT LUAD cell lines were less sensitive to these drugs than the WT cells (Figure 4). These drugs mainly targeted HDAC, PI3K and other pathways, suggesting that CTNNA2-MT cells may have low expression of proteins in these pathways. These results provide valuable guidance for the clinical treatment of patients with CTNNA2-MT LUAD.

### Enrichment of Tumor-Related Pathways After Catenin Alpha-2 Mutation

**FIGURE 5:**
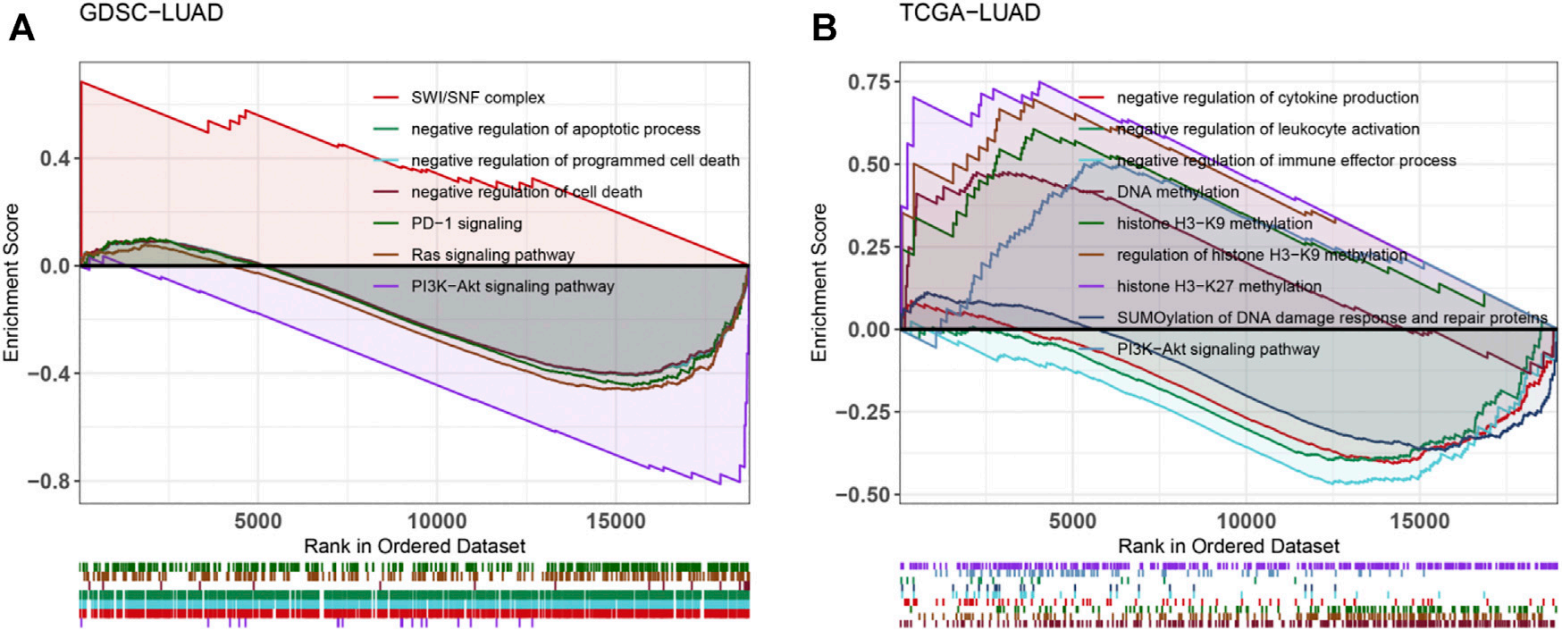
Enrichment of tumor-related pathways after CTNNA2 mutation. **(A)** Pathway enrichment analysis of differentially expressed genes between the CTNNA2-WT and CTNNA2-MT LUAD patients in the LUAD cohort of TCGA. **(B)** Pathway enrichment analysis of differentially expressed genes between the CTNNA2-WT and CTNNA2-MT LUAD cell lines in the GDSC database.

We conducted GSEA analysis on the differentially expressed genes between CTNNA2-WT patients and CTNNA2-MT patients in the LUAD cohort of TCGA (Figure 5A). The results showed that DNA methylation-and DDR-related pathways were enriched in the CTNNA2-MT group. In the immune microenvironment, negative regulation of immune system processes, negative regulation of leukocyte activation, negative regulation of cytokine production and negative regulation of immune effector processes were enriched in the CTNNA2-WT group, indicating that after CTNNA2 mutation, negative immune regulation was decreased. We also analyzed the differentially expressed genes between CTNNA2-WT and CTNNA2-MT LUAD cell lines in the GDSC database (Figure 5B). The results showed that negative regulation of cell death, negative regulation of the apoptotic process, negative regulation of programmed cell death and PD-1 signaling were enriched in the CTNNA2-WT group, indicating that after CTNNA2 mutation, negative regulators of cell death and cell damage decreased. In addition, both of these findings indicate that the PI3K-Akt signaling pathway was enriched in the CTNNA2-WT group. According to reports, the PI3K pathway is often overactivated in malignant tumors, and inhibition of the PI3K pathway can enhance the antitumor activity of the immune system (Collins et al., 2018; Borcoman et al., 2019). This result is consistent with the low expression of the PI3K pathway, which was observed in the immune-activated CTNNA2-MT group.

### Catenin Alpha-2 Mutation was Associated with Longer Overall Survival in Patients with Lung Adenocarcinoma

**FIGURE 6:**
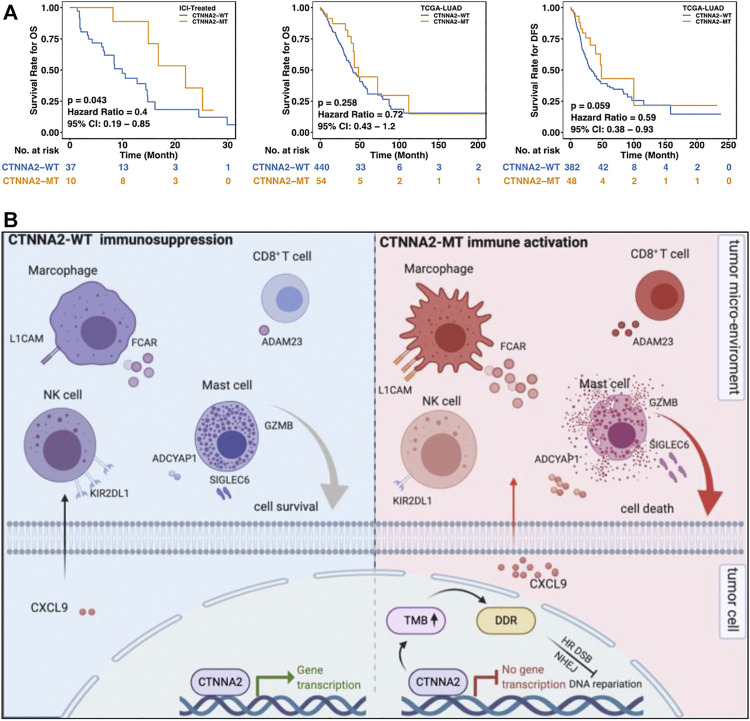
CTNNA2 mutation was associated with better survival of LUAD patients. **(A)** The left picture shows the Kaplan–Meier survival curve of OS in the CTNNA2-WT (*n* = 37) and CTNNA2-MT (*n* = 10) LUAD patients in the ICI-treated cohort. The middle image shows the Kaplan–Meier survival curve of OS in the CTNNA2-WT (*n* = 440) and CTNNA2-MT (*n* = 54) LUAD patients in the LUAD cohort of TCGA. The picture on the right shows the Kaplan-Meier survival curve of DFS in the CTNNA2-WT (*n* = 382) and CTNNA2-MT (*n* = 48) LUAD patients in the LUAD cohort of TCGA using the log-rank test. **(B)** The mechanism by which CTNNA2 mutations cause changes in the immune microenvironment of patients with LUAD.

We performed prognostic analysis of LUAD patients in the ICI-treated cohort and LUAD cohort of TCGA. The results showed that patients with CTNNA2 mutation had a longer OS than those without CTNNA2 mutation in the ICI-treated cohort (log-rank test, *p* = 0.043). The LUAD cohort of TCGA also showed a trend of longer OS in CTNNA2-MT patients (log-rank test, *p* = 0.258), and the trend of longer disease-free survival (DFS) was also observed in CTNNA2-MT patients (log-rank test, *p* = 0.059) (Figure 6A). To analyze the relationship between CTNNA2 mutation and prognosis in NSCLC and LUAD patients without immunotherapy, we used the MSKCC cohort and found that CTNNA2 mutation had no effect on progression-free survival (PFS) (Supplementary Figures S4A,B). We also used the TRACERx cohort for analysis in NSCLC patients, and the results showed no difference in OS between CTNNA2-WT patients and CTNNA2-MT patients (Supplementary Figure S4C). In addition, the CTNNA2 mutation has been shown to be associated with the prognosis of gastric cancer, laryngeal cancer, and pancreatic ductal cancer (Fanjul-Fernandez et al., 2013; Wang et al., 2014; Rizzato et al., 2016). These results indicate that CTNNA2 can be used as a predictor of prognosis in patients with LUAD. The mechanism by which CTNNA2 mutations cause changes in the immune microenvironment of patients with LUAD (Figure 6B).

## Discussion

The tumor microenvironment of LUAD affects the survival of tumor cells and the prognosis of tumor patients. Some papers have confirmed that activation of the immune microenvironment is beneficial to the prognosis of tumor patients. As a link protein, CTNNA2 participates in the occurrence and development of many diseases, and CTNNA2 protein is closely related to the occurrence and development of tumors. Our results confirmed that the overall survival of LUAD patients with CTNNA2 mutation was longer than that of those with CTNNA2 mutations and that CTNNA2 mutations were related to TMB. To our surprise, analysis of the cohort of CGA showed that patients with CTNNA2 mutation had more tumor neoantigens, suggesting that this mutation might play a critical role in the tumor immune microenvironment. We hypothesized that CTNNA2 mutation can increase the transcription level of GZMB and CXCL9, causing changes in the immune microenvironment. These changes in the immune microenvironment include elevated expression of FCAR and L1CAM receptors on macrophages, increased secretion of ADAM23 by CD8+ T cells and increased secretion of ADCYAP1 by mast cells. Changes in the immune microenvironment affect the survival of tumor cells and prolong the survival period of patients with LUAD. Our results not only confirm the role of CTNNA2 mutation in LUAD but also suggest that the effect of CTNNA2 may occur *via* changes to the immune microenvironment, providing strong evidence for CTNNA2 as a new tumor microenvironment therapeutic target.

Papers have shown that CTNNA2 is closely related to the treatment and prognosis of many tumors. CTNNA2 is a tumor suppressor gene frequently mutated in laryngeal cancer, and CTNNA2 and CTNNA3 mutations increase the migration and invasion capabilities of head and neck squamous cell carcinoma cells (Fanjul-Fernandez et al., 2013; Wang et al., 2014; Rizzato et al., 2016). Novel-miR-4885 can bind to the 3’ untranslated region of CTNNA2, thereby reducing cell adhesion and promoting the epithelial-mesenchymal transition of esophageal cancer cells. The relationship between LUAD and the immune microenvironment is inseparable. Many papers have shown that the immune microenvironment has an important effect on the prognosis of patients with LUAD. For example, CD8+ T cell infiltration leads to a better prognosis, while neutrophil and NK cell infiltration leads to a worse prognosis. Cox risk analysis showed that increased neutrophil infiltration is an independent risk factor for poor prognosis (Chen et al., 2019). This article discusses the changes in immune cell genes after CTNNA2 mutation and reveals the impact of CTNNA2 mutation on the immune environment in patients with LUAD. Patients with LUAD can be divided by immunodeficiency subtype and immune activation subtype (Seo et al., 2018), which also played a guiding role in the research presented in this article. In addition, changes in the immune microenvironment resulting from CTNNA2 mutation in other tumors warrant further study.

The underlying feature of cancer is genome instability, so therapies targeted against the DDR have great potential. Inhibitors based on the DNA damage response, including PARP, ATR, ATM, CHEK1, CHEK2, DNAPK and WEE1 inhibitors, all play a major role in the treatment of tumors, especially BRCA-mutant tumors (Pilié et al., 2019). It has also been found that in LUAD, the regulation of the DDR can affect the response of tumor cells to radiation (Dong et al., 2019). This article also found that the number of DDR mutations is upregulated after CTNNA2 mutation in patients with LUAD, indicating that treating these patients with a DDR inhibitor may improve their prognosis. In addition, drug sensitivity data showed that CTNNA2-MT cell lines are more resistant than WT cells to HDAC, EGFR, and PI3K inhibitors, suggesting that CTNNA2-MT cell lines may exhibit decreased expression of the proteins in these pathways. It has been reported that the role of HDAC in LUAD is closely related to EZH2. NSCLC patients with high expression of HDAC and EZH2 have a low survival rate (Shi et al., 2019), and EGFR and PI3K are classic regulatory pathways involved in the development of LUAD (Wei et al., 2019; Shaurova et al., 2020). This motivates further exploration and clinical application of these potential targets.

## Data Availability

The original contributions presented in the study are included in the article/Supplementary Material, further inquiries can be directed to the corresponding authors.
