# Supplementary figures and images for: Catenin Alpha-2 Mutation Changes the Immune Microenvironment in Lung Adenocarcinoma Patients Receiving Immune Checkpoint Inhibitors
Source: Front Pharmacol. 2021 Jun 7;12:645862. doi: 10.3389/fphar.2021.645862 (PMC8215613; doi:10.3389/fphar.2021.645862)

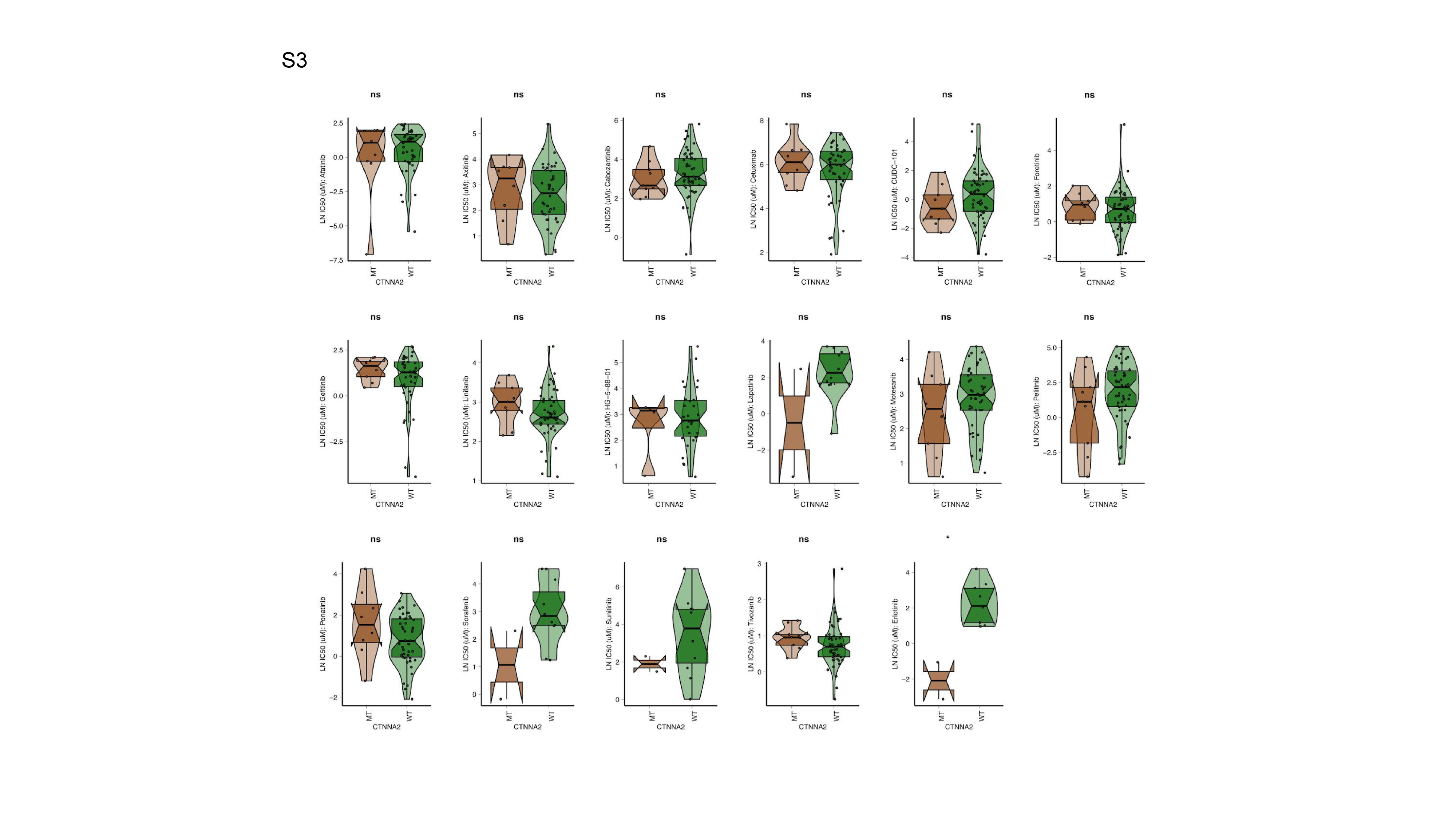

Supplement: Supplementary file 3 [file Image3.TIF]

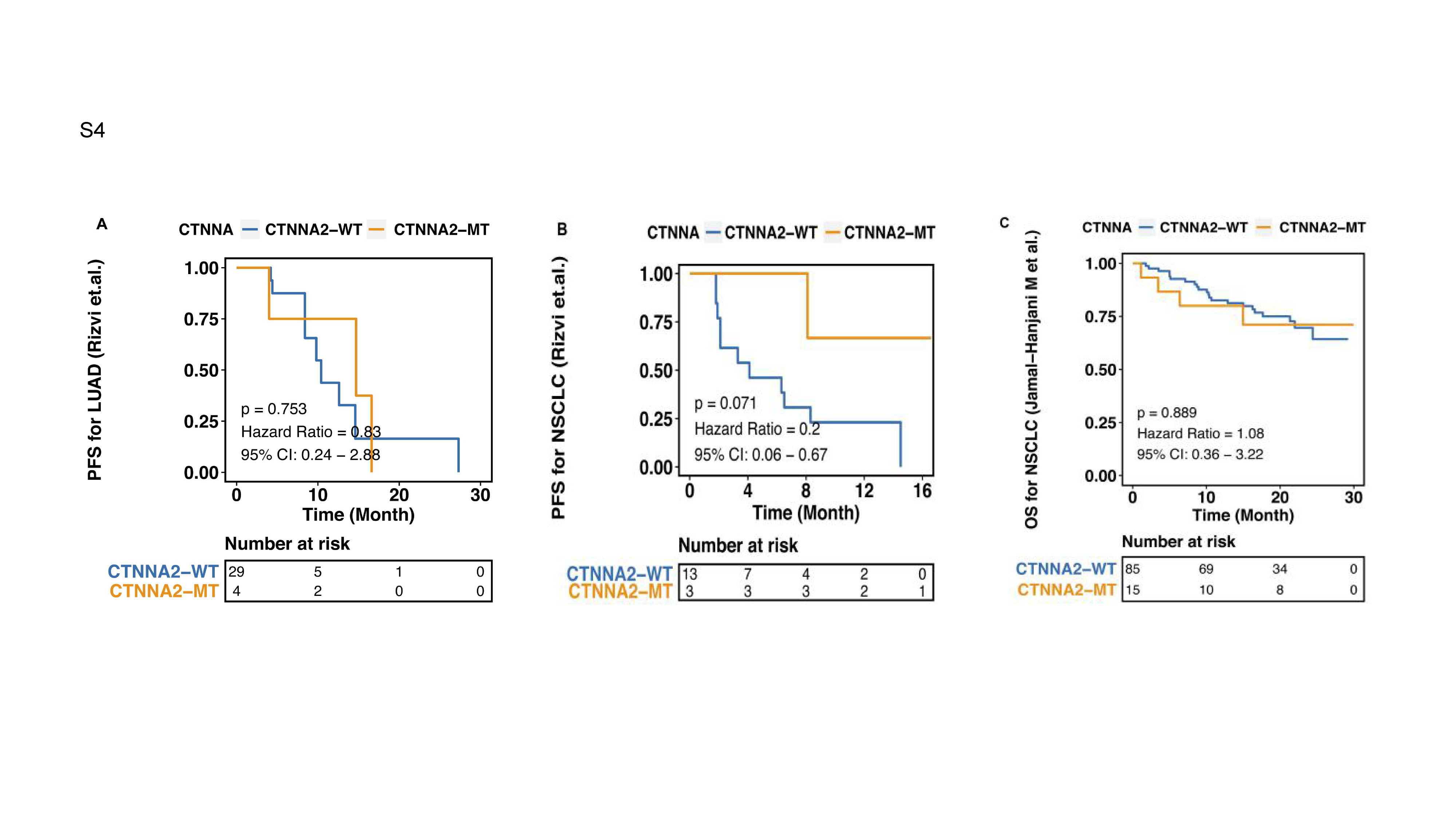

Supplement: Supplementary file 4 [file Image4.TIF]

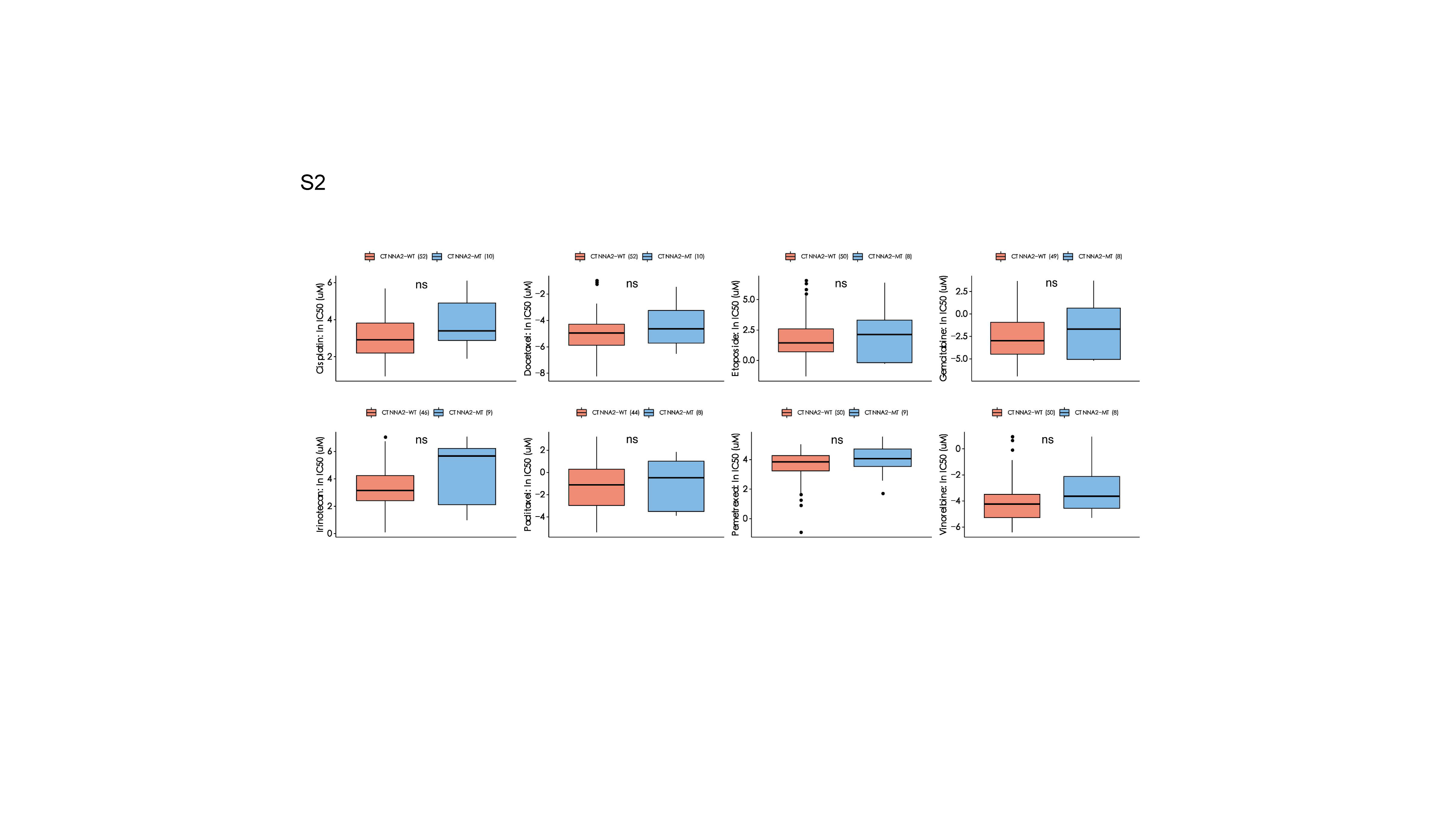

Supplement: Supplementary file 5 [file Image2.TIF]

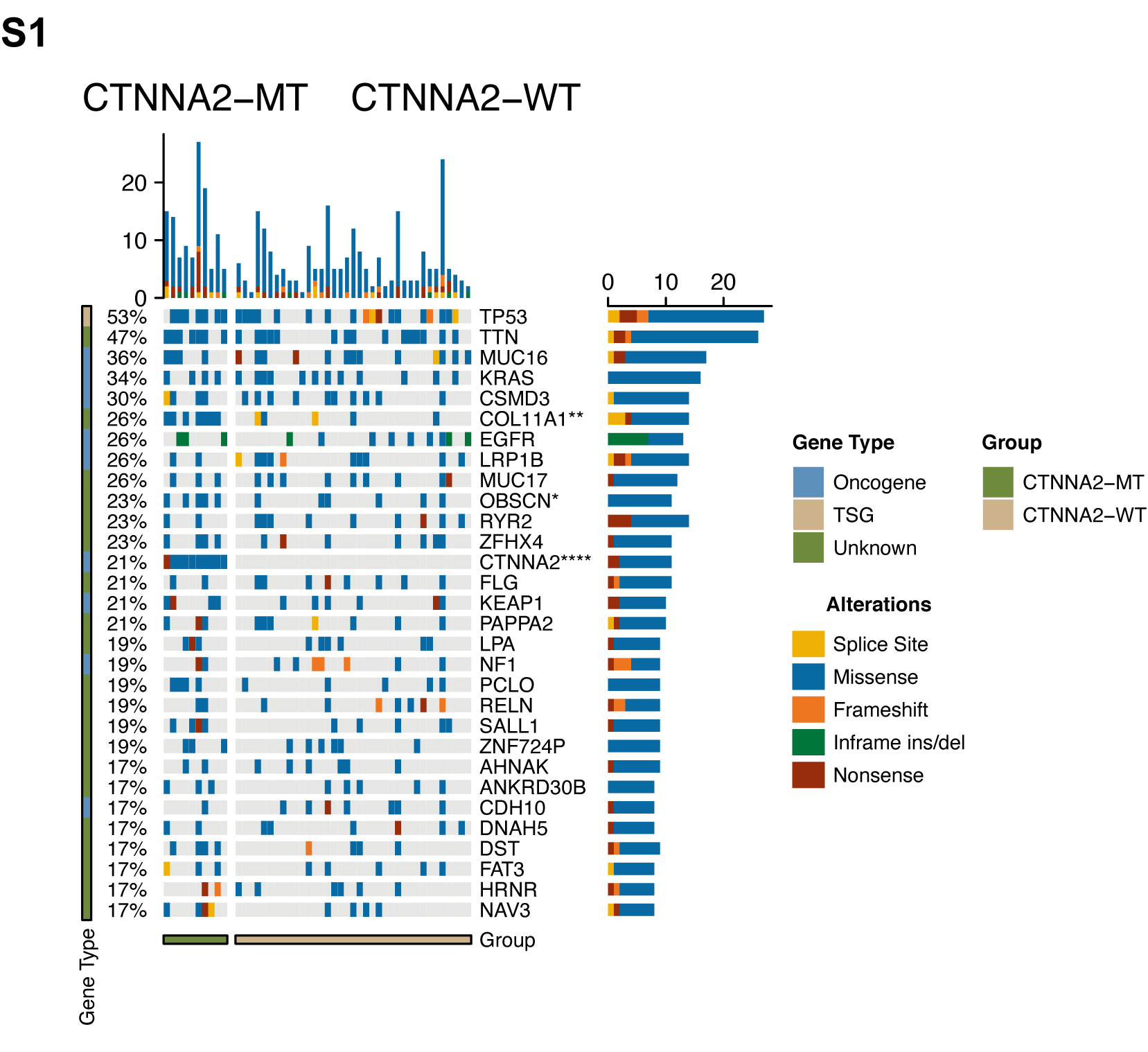

Supplement: Supplementary file 6 [file Image1.TIF]
